# Pulpless Teeth

**Published:** 1885-12

**Authors:** Chas. F. Ives

**Affiliations:** New York.


ARTICLE III.

### PULPLESS TEETH.

BY CHAS. F. IVES, M. D. S., NEW YORK.

I have no intention of going over any portion of the ground so repeatedly and thoroughly trodden by others on this subject, but rather to offer a few thoughts which have been suggested to my mind after reading the articles in the *Medical Record* and listening to remarks from different members of the dental profession in reply.

Entirely agreeing with the verdict that physicians generally are far from being well posted in diseases of the teeth,

I still could not help wondering *why* these articles were written—what motive prompted them. Surely it was not from any desire to snub us as a profession—not to gratify any personal antipathy, and not altogether the result of clinical observations. Back of all this there must have been some honest convictions which led to the attack. Perhaps, a succession of unpleasant experiences, the result of slow and wearisome endeavors to find a cause for certain pathological conditions, the tracing of which through avenues leading to one common center, resulted in certain convictions, convictions so strong as to overlook the fact that under other conditions than those existing, the result might have been different. For instance, if in their private practice they met with cases which baffled them in diagnosis and treatment, and after having almost exhausted their skill in the vain attempt to get at the *cause*, they at last get at the *fact*, that there is at times, an uneasiness, an occasional approach to pain, sufficient to be acknowledged if persistently questioned in regard to it, and which seems to lie in or about the oral cavity. This general uneasiness is at last traced to a pulpless tooth, which has been treated and filled, or quite as often, to one in which the attempt has been made to cap an exposed pulp, or a metal filling was laid in too close proximity to it. Extraction of the tooth at once removes all the troublesome symptoms for the alleviations of which the physician was consulted. A few experiences of this kind would naturally lead many physicians to the conclusion that teeth deprived of their pulps were very like slivers in the fingers, foreign bodies, and the sooner they were removed the better. I have more than once heard this assertion from the medical fraternity. Can we, looking at it in this light, conscientiously blame them? We *know* that if pulpless teeth are brought to a healthy condition, and their roots filled as well as it is possible to do it, the cases in which trouble supervenes are reduced to a minimum, and there is no necessity for consultation with the physician in regard to any trouble for which they are responsible. Now the point I am after is this. What proportion of pulpless teeth are honestly, conscientiously and intelligently treated and filled by the dentist? Of how many of these teeth coming to him for treatment can he say, after finishing his work, I have done my best regardless of time or remuneration. This is the serious side of the subject for us. To further illustrate let me give the history of a single case.

A gentleman on his way to have a first superior molar extracted, suddenly thought better of it. and came to me for temporary relief, which having afforded him, he volunteered the following statement in regard to it. Some four years before, he put himself in the hands of a dentist in New York, who found in this tooth, an exposed pulp which he destroyed in the usual way. The tooth had a large gold filling in its crown, but the application for destroying the pulp, and the subsequent treatment was made through a cavity in the posterior approximal surface. In due time, the dead pulp was removed from its chamber, and attempts made to enter the root canals. The palatal root offered but little difficulty apparently, but the buccal roots proved more of a task, and after many vain attempts, the dentist assured him that they were entirely closed with a deposit of secondary dentine. The palatal root was dressed, and a temporary stopping inserted. From this time on the tooth was troublesome, a continuous nagging uneasiness was present which no applications seemed to relieve. Sometime after this, the patient passed into the hands of another dentist, who drilled through the crown filling, thus obtaining more direct access to the roots. Treatment was resumed with but little improvement, and the tooth was finally filled with gutta percha, with the advice to have it extracted if it proved troublesome. This was its history when he came into my hands. I removed the entire filling in the crown, enlarged the opening until I had perfectly free access to all of the roots. I found the entrance to both buccal roots, enlarged them and followed them up till their extreme minuteness and entire freedom from odor convinced me that there would be no further trouble from them. The palatal root was in a bad condition and undoubtedly the seat of the whole trouble, as the end of it had never been reached. It was stubborn under treatment, and carbolic acid or iodiform left in it for a few days would thoroughly lose its identity. I therefore without hesitation went through the foramen with a drill made from a Donaldson bristle. A call for pus was made with the peroxide of hydrogen, and promptly responded to and from that time on it has continued to improve, and will, I trust, soon be in condition to fill permanently. Well, what of it? Simply this: Do you not honestly believe that if the dentist who destroyed the pulp in that tooth, had removed the crown filling, obtained free access to the roots, given them such treatment as was necessary and filled them, it would probably never have given trouble? Could we naturally look for success to follow the endeavor to reach the anterior buccal root of a superior molar through a posterior approximal cavity, or to do effectual service in any of them.

It is just such kind of imperfect operations as this that send not a few to the physicians with suspected neuralgias and other neuroses. It is the results of just such operations that occasionally get reported in the medical journals, with an editorial fling at the end to the effect that "Dentists should remember that the treatment of diseased tissues require a medical education." I have seen during the past winter enough of careless, unprincipled work performed on this class of teeth, by men who claim to stand high in the profession, to warrant wholesale denunciation. The time is coming when the cry of mal-practice will mean business to those who are unwilling to spend the time necessary on diseased teeth, because it does not bring them the same remuneration as ordinary operations.

It needs a great deal of patience, a great deal of good temper, and a large amount of persistent determinative energy to successively treat a refractory pulpless tooth.

No operation draws more heavily on the nervous system, for it often discourages while it wearies, and it is at such times that the tempter draws near, and one needs all his strength to say "get thee behind me." If we are unwilling to give our best efforts in this special line of work, if we believe that it does not pay to devote the time and thoroughness to it necessary for the best results, then let us believe that the physicians are right, and that extraction is the best thing, and not stand up and pity their ignorance. If on the other hand we strive with all honesty of purpose to do the best we are capable of doing, we assuredly shall prove, in a large degree, successful; and not only enjoy the consciousness of a duty well done, but be able to convince our medical friends that pulpless teeth can be made very quiet and respectable members of oral society though slightly crippled.

Before concluding I cannot resist having my little say in regard to the different materials used in the filling of root canals. In a practice of twenty-five years I have naturally had occasion to remove quite a number of root fillings, re-treat and re-fill them, and whenever I have had the privilege of obtaining an extracted tooth with the roots filled, I have sat down with keen delight to a careful dissection of it, hoping to profit from the success or failure of the operator. I have never yet, in the root or roots of a tooth, in or out of the mouth, found a filling of gutta-percha, metal of any kind, wood or paste, that did not on removal give out more or less of vile odor. That it is possible to fill a larger proportion of roots more perfectly—so far as adaptation is concerned—with a solution of gutta-percha in chloroform than with any other material, I am inclined to believe; but that it can be made impervious to emanations from the tubuli or through the foramen, I do *not* believe. The fact that those who use it and claim that they never have any trouble, is no proof to the contary. I had occasion some time ago to re-fill the crowns of two superior bi-cuspids, the roots of which had been filled with cotton and creosote some thirteen years before. They had never given the slightest trouble, but my curiosity getting the better of me I removed the fillings,finding it,however,no easy task,for they had evidently been packed on the principal of a cotton bale. As each bit was removed, it was passed in close proximity to my nasal organ, and to the very end it was sweet and redolent of the antiseptic. I therefore put it down that cotten and creosote could be *made* to answer a good purpose, and in this case back it went. In my hands nothing has been so uniformly successful as a slow setting oxy-chloride of zinc, mixed to a moderate degree of stiffness and with a fibre or two of cotton to make it carry well; first closing the foramen with a bit of cotton moistened with carbolic acid, or if the opening is large, with tin foil. With this, a root can be thoroughly filled and rendered aceptic to any secretions. I have no sympathy with that mode of practice which goes upon the principal that all root fillings should be tentative in their character, and therefore filling them in a manner and material to be easily removed in case of trouble arising, or providing a tap or vent-hole. I would far rather bend all my energy to get the roots in a healthy condition, and when my judgment assured me they were so, fill them as I would a cavity, to the best of my ability and never stop to think that they would do otherwise than well.

M. L. Rhein: There is but one point in the paper that I wish to speak of, and that is in regard to what the essayist says about gutta-percha fillings. I believe a root can be perfectly filled with gutta-percha and neither cause trouble nor give forth the slightest odor. I have removed many a gutta-percha filling and have yet to detect any odor. I know that this depends a great deal upon the manner in which the root is filled, but I think gutta-percha better in any case than anything else that can be used. To fill a root with gutta-percha it is necessary to reach the very apex, and to know that the root is in a perfectly healthy condition—be convinced that there is no further trace of putrefaction. I then introduce a little alcohol into the cavity, and having my lamp handy, I dry the root out very thoroughly by means of the hot-air syringe, so that it is warm, and then I introduce the gutta-percha solution upon a platinum point. The canal is in this way thoroughly dried and the tubuli are in condition to take up the chloroform solution. I fill about half the root in that way, and the tooth temporarily with oxy-phosphate.

I do not mean to say that other materials will not perfectly fill teeth roots. I suppose there are a hundred ways of doing it successfully; but I am sure that gutta-percha can be used in a root, and the operation made a success, and I don't want it to go out that this is not the case.

Chas. F. Ives: I believe I stated that other material could be used and as perfectly as that to which I gave the preference; but you cannot make a gutta-percha filling that will not absorb; it will stink to the end of time.

M. L. Rhein: Where would it absorb, through the apex?

Chas. F. Ives: Through the apex and tubuli.—*Dental Society of State of N. Y.—Odontographic Journal.*

